# Do Hernias Contribute to Increased Severity of Aneurysmal Disease among Abdominal Aortic Aneurysm Patients?

**DOI:** 10.1055/s-0040-1719113

**Published:** 2021-06-03

**Authors:** Irene Hinterseher, Milena Miszczuk, Florian Corvinus, Carolin Zimmermann, Mariana Estrelinha, Diane T. Smelser, Helena Kuivaniemi

**Affiliations:** 1 Vascular Surgery Clinic, Charité—Universitätsmedizin Berlin, corporate member of Freie Universität Berlin, Humboldt-Universität zu Berlin, and Berlin Institute of Health, Berlin, Germany; 2 Department of General, Visceral and Transplant Surgery, Universitätsmedizin Mainz, Mainz, Germany; 3 Department of Visceral, Thoracic and Vascular Surgery, University Hospital Carl Gustav Carus, Technical University of Dresden, Dresden, Germany; 4 Clinic of Vascular and Endovascular Surgery, Ludwigsburg, Germany; 5 Sigfried and Janet Weis Center for Research, Geisinger Health System, Danville, Pennsylvania; 6 Division of Molecular Biology and Human Genetics, Department of Biomedical Sciences, Faculty of Medicine and Health Sciences, Stellenbosch University, Tygerberg, South Africa

**Keywords:** abdominal aortic aneurysm, hernia, connective tissue disorder

## Abstract

**Background** Connective tissue disorders could contribute to the pathogenesis of both abdominal aortic aneurysms (AAA) and hernias. We tested the hypothesis that hernias in AAA patients contribute to increased severity of the aneurysmal disease.

**Methods** A questionnaire was used to collect information from 195 AAA patients divided into four groups: (1) survivors ( *n* = 22) of ruptured AAA, (2) patients ( *n* = 90) after elective open repair, (3) patients ( *n* = 43) after elective endovascular repair (EVAR), and (4) patients ( *n* = 40) under surveillance of AAA. The control group consisted of 100 patients without AAA whose abdominal computed tomography (CT) scans were examined for the presence of hernias. Mann–Whitney *U* -test, Chi-squared ( *χ* ^2^ ) test, or Fisher's exact test (as appropriate) were used for statistical analyses. Multivariate logistic regression was used to control for potential confounding variables such as sex and age.

**Results** The prevalence of inguinal hernias was significantly higher in the AAA than the control group (25 vs. 9%, *p* = 0.001) and did not differ between the AAA subgroups (9, 24, 35, and 23% in subgroups 1 through 4, respectively, *p* = 0.15) based on univariate analysis. The prevalence of inguinal hernias did not differ ( *p* = 0.15) between the two open surgery groups (groups 1 and 2), or when comparing all three operative procedures as a combined group to group 4 ( *p* = 0.73). The prevalences of incisional hernias were 18 and 24% for groups 1 and 2, respectively, with no significant difference ( *p* = 0.39). Inguinal hernia demonstrated a significant association with AAA on multivariate analysis ( *p* = 0.006; odds ratio [OR] = 4.00; 95% confidence interval [CI] = 1.49–10.66).

**Conclusions** Our study confirms previous observations that patients with AAA have a high prevalence of hernias. Our results suggest that hernias do not contribute to increased severity of the aneurysmal disease.

## Introduction

Abdominal aortic aneurysm (AAA) is a complex disease of the aging population that affects up to 10% of Caucasian males over 65 years of age and is a leading cause of death in the United States with approximately 7,000 deaths per year. 1 Due to improved diagnostic efforts using ultrasonography imaging, AAAs can be identified earlier. Despite improved diagnostic imaging, 15% of cases present as ruptured AAA in hospitals of industrialized nations. 2

AAA has several recognized risk factors such as smoking, male sex, age over 65 years, and positive family history. 3 Additionally, an increased prevalence of AAA was described in patients with pulmonary emphysema, 4 and patients with AAA have a 2.3- to 3-fold increased risk of developing inguinal hernia compared with patients with aortoiliac occlusive disease (AOD). 5 6 It is well known that mechanical factors, such as weakening of the abdominal wall after a transperitoneal operation and increased intra-abdominal pressure, increase a person's risk for hernias. Other known risk factors for inguinal hernias include male sex, age over 65 years, smoking, pulmonary emphysema, and family history. 7 Incisional hernias occur in 5 to 15% of patients undergoing transperitoneal operations. 8 Risk factors for incisional hernias include malnutrition, malignancy, wound infection, chronic obstructive pulmonary disease, diabetes mellitus, renal insufficiency, obesity, smoking, and older age. 8 Thus, AAA patients and patients with the most common forms of hernias of the abdominal wall share many risk factors.

Vascular surgeons are aware of the high risk of developing an incisional hernia after open transabdominal AAA surgery. Furthermore, AAA patients often present with large inguinal hernias or have a history of an inguinal hernia repair. 4 6 The prevalence of inguinal and incisional hernias among AAA patients was investigated in studies summarized in Tables 1 and 2 . A meta-analysis on several of these studies concluded that the prevalences of inguinal and incisional hernias among AAA patients were 23 and 21%, respectively. 6 A large retrospective cohort study found that the combined prevalence of all types of abdominal wall hernias was 16.7% in AAA patients ( *n* = 939) compared with 9.9% in the control group of peripheral arterial disease (PAD) patients ( *n* = 3,465; *p* < 0.0001). 9

**Table 1:** Review of literature on association between inguinal hernia and abdominal aortic aneurysms

Study	Examination method	Inguinal hernia a		*p* -Value b
		AAA *n* (%)	Controls *n* (%)	
Cannon et al 27	Clinical	88/341 (26)	61/417 (16)	**<0.001**
Lehnert and Wadouh 28	Clinical	49/119 (41)	15/81 (16)	**0.001**
Hall et al 29	Clinical	28/128 (22)	11/65 (17)	NS
Adye and Luna 30	Clinical	11/58 (19)	2/42 (5)	0.037
Wright and O’Dwyer 31	Clinical	25/149 (17)	–	–
Musella et al 32	MRI	20/51 (39)	13/63 (20)	**0.01**
Papadimitriou et al 33	Clinical	21/63 (33)	6/58 (10)	**<0.01**
Raffetto et al 34	Clinical	42/177 (24)	5/82 (6.1)	**0.002**
Golledge et al 4	Questionnaire	266/873 (31)	2,883/1,0872 (27)	0.01
Henriksen et al 35	Clinical	85/601 (14)	2,936/1,8331 (16)	0.632
Gindera et al 36	CT	60/236 (25.4)	26/236 (11)	**<0.001**
Müller et al 10	CT	16/99 (16.2)	9/100 (9)	0.14
Current study	Questionnaire (AAA), CT (controls)	48/195 (25)	9/100 (9)	**0.001**

Abbreviations: AAA, abdominal aortic aneurysms; CT, computed tomography; MRI, magnetic resonance imaging; NS, not significant.

a The numbers indicate the number of patients with hernia/total number of patients examined in each group.

b Comparison of hernia prevalences between AAA cases and controls, *p* -values are taken directly from the original publications.

**Table 2:** Review of literature on association between incisional hernia and abdominal aortic aneurysms

Study	Examination method	Incisional hernia a		*p* -Value b
		AAA *n* (%)	Controls (PAD) *n* (%)	
Darke and Eadie 37	Clinical	9/34 (26)		
Stevick et al 38	Clinical	10/27 (37)	4/39 (10)	**<0.01**
Hall et al 29	Clinical	13/128 (10)	2/65 (3.0)	**<0.05**
Johnson et al 39	Clinical	14/281 (5.0)	–	–
Holland et al 40	Clinical	13/34 (38.2)	6/30 (20)	NA
Adye and Luna 30	Clinical	18/58 (31)	5/42 (12)	0.025
Johnson et al 39	Clinical	26/265 (9.8)	10/331 (3.0)	NA
Musella et al 32	MRI	16/51 (32)	11/63 (17)	**<0.03**
Papadimitriou et al 33	Clinical	7/63 (11)	2/58 (3.4)	**<0.05**
Raffetto et al 34	Clinical	50/177 (28)	9/82 (11)	**0.003**
Liapis et al 41	Clinical	32/197 (16)	5/67 (7.4)	0.029
Rodriguez et al 42	CT	14/61 (23)	–	–
Fassiadis et al 43	Clinical + US	26/37 (79)	–	–
Laohapensang et al 24	Clinical	4/18 (22)	–	–
Panayiotopoulos et al 44	NA	134/421 (32)	–	–
Bevis et al 45	Clinical + US	16/43 (37)	–	–
Gruppo et al 46	Clinical	51/412 (12)	73/653 (11)	0.62
Henriksen et al 21	Clinical	108/1759 (6.1)	40/838 (4.7)	0.16
Wiegering et al 47	Clinical questionnaire	10/72 (13.9)	7/27 (25.9; colorectal surgery)	NS
Current study	Questionnaire	26/112 (23)	–	–

Abbreviations: AAA, abdominal aortic aneurysms; MRI, magnetic resonance imaging; NA, data not available; NS, not significant; PAD, peripheral arterial disease; US, ultrasonography

a The numbers indicate the number of patients with hernia/total number of patients examined in each group.

b Comparison of hernia prevalence between AAA cases and controls, *p* -values are taken directly from the original publications.

The main aim of this study was to determine if hernias contribute to increased severity of the aneurysmal disease. The underlying hypothesis was that AAA is a connective-tissue disorder demonstrating vascular weakness and therefore more advanced stages of the AAA disease (i.e., diameter > 5 cm or ruptured AAA) are associated with a higher prevalence of hernias.

## Materials and Methods

### Patient Groups and Data Collection

A questionnaire was designed to collect the following information: (1) presence of inguinal and incisional hernias, (2) history of hernia operations, and (3) family history of AAA. The questionnaire was sent to 249 AAA patients treated in the Vascular Center of the University Hospital Carl Gustav Carus, Technical University of Dresden, Germany, from 1995 to 2005. The questionnaire was administered from September to December 2006. The study was approved by the Ethics Committee of the Medical School at the Technical University of Dresden, Germany (EK346112010). According to the institutional guidelines, informed consent for the retrospective data analysis was waived. The questionnaire was completed by 195 (78%) of the 249 patients. The 195 AAA patients were divided into four groups based on the treatment. The groups reflected the stage and severity of the aneurysmal disease: (1) survivors after open repair of ruptured AAA (rAAA; 22 completed the questionnaire out of 26 who received the questionnaire; participation rate: 85%), (2) patients ( *n* = 90/98; 92%) who had undergone elective open repair (eAAA), (3) patients ( *n* = 43/47; 91%) who had elective endovascular treatment (EVAR), and 4) patients ( *n* = 40/78; 51%) with a small AAA under surveillance (sAAA).

Open surgical repair was performed by transperitoneal approach with midline incision. Fascial closure was done for all patients with continuous absorbable suture by experienced vascular surgeons who are all also certified general surgeons. Elective open and endovascular repair of AAA was performed if the aortic diameter was ≥ 5.0 cm. Small AAAs under surveillance had a diameter of 3.0 to 4.9 cm and were diagnosed by ultrasonography.

Clinical data (prior to the beginning of the study) for the AAA patients were collected from the medical records, and all laboratory measurements were preoperative.

A control group ( *n* = 100) consisted of patients with no history of AAA (infrarenal diameter <3 cm) and no other aneurysms. All control patients underwent an abdominal computed tomography angiography (CTA) scan at the Department of Radiology, Charité, Berlin, Germany, in 2005 to 2014 either for evaluation of a kidney donation ( *n* = 14) or as part of screening for melanoma recurrence ( *n* = 86). We calculated the age at the time of the CTA scan. The CTA scans were evaluated for presence of hernia, and patients' clinical data were collected from the medical records as previously described. 10

### Statistical Analyses

First, we performed a univariate comparison of the AAA and control group. The mean, median, standard deviation (SD), maximum, and minimum values were calculated. For categorical variables we performed a cross tabulation. To measure the statistical significance, Mann–Whitney *U* –test, Chi-squared ( *χ* ^2^ ) test, and Fisher's exact test (as appropriate) were used.

The prevalences of inguinal and incisional hernias were estimated for each treatment group separately. Differences in prevalences of inguinal and incisional hernias among the four AAA patient groups were tested using *χ* ^2^ test when the sample size for each cell in the analysis was >5 or Fisher's exact test when the sample size was ≤5. Multivariate logistic regression was utilized to control for confounding variables of sex and age.

Age is reported in years at the time the questionnaire was administered (when information regarding the occurrence of a hernia was obtained). Statistical Analysis Software (SAS V.9.2 for Windows, SAS Institute Inc., Cary, NC) and SPSS statistical package (v22 for Windows, IBM, Armonk, NY) were used for analyses.

### Literature Search

All studies which determined the incidence of inguinal hernias and/or incisional hernias in patients with AAA were identified using a two-step search strategy. First, a PubMed search with keywords “aneurysm AND hernia” was performed for articles published between 1966 and May 2019. Second, relevant studies were identified by a manual search in the references of the identified articles.

## Results

The AAA group consisted of 195 patients, with a mean age of 75.9 ( ± 8.6) years. There was no significant difference in age among males ( *n* = 178) and females ( *n* = 17; *p* = 0.08). The control group included 100 patients with a mean age of 71.2 ( ± 8.6) years and was therefore significantly younger than the AAA group (75.9 ± 8.6; *p* < 0.001). The mean age was 76.2 ± 8.4 years for the rAAA, 75.5 ± 8.8 years for the eAAA, 75.2 ± 8.6 years for EVAR, and 76.8 ± 7.5 years for the sAAA group. The mean sizes of the diameters of the AAAs were 5.8 ± 1.1 cm for the eAAA, 5.8 ± 0.8 cm for EVAR, and 4.2 ± 0.5 cm for the sAAA group. Altogether 11 (6%) of the 195 patients reported positive family history for AAA. Among the four subgroups, the number of AAA patients with positive family history varied from one (2%) in the EVAR group to three (14%) in the rAAA group.

Clinical data from the AAA and control patients are presented in Table 3 . AAA patients were more likely to suffer from hypertension, PAD, coronary artery disease (CAD), dyslipidemia and chronic obstructive pulmonary disease (COPD; all *p* < 0.001), as well as to be ever smokers ( *p* < 0.001). Treatment with medications (aspirin, nitrates, β-blockers, and statins) was significantly higher in the AAA group. AAA patients had significantly lower hemoglobin level and thrombocyte count but higher glucose and creatinine level. In multivariate analysis, we found a significant association of AAA with hypertension, CAD, COPD, and ever smoking ( Table 4 ).

**Table 3:** Characteristics of the abdominal aortic aneurysms and control groups

Variable	AAA group *n* = 195	Control group *n* = 100	*p* -Value
Age (y) at time of study Mean ± SD	75.9 ± 8.6	71.2 ± 8.6	**<0.001**
Males (%)	91.3	79.0	**0.005**
Height (cm) Mean ± SD	171.9 ± 15.4	174.0 ± 8.1	0.245
Weight (kg) Mean ± SD	79.1 ± 14.2	79.5 ± 13.9	0.429
Hemoglobin (mmol/L) Mean ± SD	8.4 ± 1.2	14.5 ± 11.9	**<0.001**
White blood cell count (×10 ^9^ /L) Mean ± SD	7.9 ± 3.4	8.7 ± 6.1	0.644
Thrombocyte count (×10 ^9^ /L) Mean ± SD	221.7 ± 64.7	253.5 ± 94.9	0.010
Glucose Mean ± SD	6.7 ± 2.2	6.2 ± 2.4	0.015
C-reactive protein (mg/L) Mean ± SD	21.9 ± 41.1	29.3 ± 54.1	0.337
Cholesterol (mmol/L) Mean ± SD	5.3 ± 1.4	5.5 ± 0.8	0.581
LDL (mmol/L) Mean ± SD	3.5 ± 1.3	3.7 ± 0.6	0.631
HDL (mmol/L) Mean ± SD	1.2 ± 0.6	1.1 ± 0.2	0.815
Triglycerides (mmol/L) Mean ± SD	1.8 ± 0.6	2.2 ± 0.7	0.106
Creatinine (mmol/L) Mean ± SD	111.8 ± 83.3	91.9 ± 34.2	**<0.001**
Hypertension a (%)	97.8	70.0	**<0.001**
Peripheral arterial disease (%)	28.3	8.1	**<0.001**
Coronary artery disease (%)	58.6	19.0	**<0.001**
Myocardial infarction (%)	25.4	14.0	0.33
Diabetes mellitus (%)	22.1	20.4	0.879
Dyslipidemia b (%)	61.5	34.7	**<0.001**
COPD (%)	28.0	4.0	**<0.001**
Ever smoke (%)	77.2	52.9	**<0.001**
Aspirin (%)	75.7	27.4	**<0.001**
Clopidogrel (%)	5.7	7.4	0.606
Coumadin (%)	13.1	7.4	0.223
Nitrates (%)	17.7	6.3	**0.009**
Calcium antagonists (%)	27.3	17.9	0.101
ACE inhibitors (%)	47.7	38.9	0.200
Beta-blockers (%)	60.8	46.3	0.029
Statins (%)	51.4	34.7	0.010

Abbreviations: AAA, abdominal aortic aneurysms; ACE, angiotensin converting enzyme; COPD, chronic obstructive pulmonary disease; HDL, high-density lipoprotein; LDL, low-density lipoprotein; SD, standard deviation.

Note: Laboratory results are shown as mean ± SD and were measured preoperatively for AAA or at the time of the computed tomography angiography examination for the control group.

a Blood pressure ≥ 140/90 mm Hg.

b Increased LDL (male and female: ≥ 4.12 mmol/L; age > 35 years) and decreased HDL (male: ≤ 0.90 mmol/l; female ≤ 1.3 mmol/L; age > 35 years).

**Table 4:** Results of the multivariate analysis between the abdominal aortic aneurysms and control patients

Variable	OR (95% CI)	*p* -Value
Hypertension	8.13 (2.25–29.41)	**0.001**
Coronary artery disease	3.71 (1.89–7.29)	**<0.001**
COPD	6.82 (1.95–23.85)	**0.003**
Ever smoke	2.55 (1.31–4.95)	0.006
Peripheral arterial disease	1.82 (0.68–4.84)	0.232
Dyslipidemia	1.43 (0.73–2.81)	0.297

Abbreviations: CI, confidence interval; COPD, chronic obstructive pulmonary disease; OR, odds ratio.

Clinical data for each AAA subgroup is presented in Table 5 . We looked at the age of the patients at two time points, at the initiation of the study and at the time of operation. Age did not differ significantly between the groups for either time point. Cardiovascular risk factors between the groups were not significantly different, with the exception of dyslipidemia, which was significantly lower in the rupture group. Treatment with medications (statins and β-blockers) was significantly lower in the ruptured AAA group. Preoperative laboratory measurements for C-reactive protein were significantly higher in patients with ruptured AAA. A comparison of each AAA group with the control group did not reveal any substantial differences, as stated in Table 6 .

**Table 5:** Characteristics of the abdominal aortic aneurysms groups

Variable	rAAA (open) ( *n* = 21)	eAAA (open) ( *n* = 90)	EVAR ( *n* = 42)	sAAA ( *n* = 39)	*p* -Value
Age (y) at time of study Mean ± SD	76.2 ± 8.4	75.5 ± 8.8	75.2 ± 8.6	76.8 ± 7.5	0.83
Age (y) at operation Mean ± SD	70.2 ± 8.2	68.4 ± 8.7	71.1 ± 9.0	N/A	0.12
Males (%)	85.7	91.1	97.6	87.2	0.22
Height (cm)	169.9 ± 9.1	171.0 ± 20.4	173.5 ± 6.3	175.3 ± 7.3	0.16
Weight (kg)	72.6 ± 12.5	79.4 ± 16.3	80.2 ± 10.8	81.9 ± 11.8	0.07
Hemoglobin (mmol/L) Mean ± SD	7.5 ± 1.4	8.5 ± 0.5	8.6 ± 1.0	8.5 ± 0.9	**<0.01**
White blood cell count (× 10 ^9^ /L) Mean ± SD	9.9 ± 3.0	7.2 ± 1.8	7.4 ± 1.7	10.3 ± 8.8	**< 0.01**
Thrombocyte count (× 10 ^9^ /L) Mean ± SD	221.7 ± 87.1	223.9 ± 61.1	219.4 ± 67.0	214.8 ± 39.2	0.96
Glucose Mean ± SD	7.8 ± 3.2	6.3 ± 1.7	6.6 ± 2.0	7.4 ± 3.6	0.06
C-reactive protein (mg/L)	94.8 ± 63.5	9.3 ± 16.3	10.9 ± 16.8	6.6 ± 9.4	**<0.01**
Cholesterol (mmol/L) Mean ± SD	4.8 ± 1.5	5.5 ± 1.3	5.2 ± 1.4	4.8 ± 1.5	0.28
LDL (mmol/L) Mean ± SD	3.4 ± 2.0	3.7 ± 1.3	3.5 ± 1.1	3.5 ± 1.0	0.76
HDL (mmol/L) Mean ± SD	0.9 ± 0.4	1.2 ± 0.6	1.6 ± 10.7	1.2 ± 0.4	0.28
Triglycerides (mmol/L) Mean ± SD	1.8 ± 0.7	1.7 ± 0.6	1.9 ± 1.4	1.8 ± 0.9	0.84
Creatinine (mmol/L) Mean ± SD	100.7 ± 27.1	114.4 ± 107.0	105.7 ± 33.8	129.0 ± 78.5	0.74
Diameter (cm) Mean ± SD	NA	5.8 ± 1.1	5.8 ± 0.8	4.2 ± 0.5	**<0.01**
Hypertension a (%)	95.2	96.5	100	100.0	0.36
Peripheral arterial disease (%)	42.9	27.9	22.0	28.1	0.39
Coronary artery disease (%)	47.6	53.5	73.8	59.4	0.11
Myocardial infarction (%)	14.3	24.1	29.3	30.3	0.54
Diabetes mellitus (%)	23.8	18.6	26.8	23.8	0.73
Dyslipidemia b (%)	33.3	59.6	68.3	77.4	**0.01**
COPD (%)	23.8	28.2	26.8	28.6	0.98
Ever smoke (%)	80.0	70.2	90.0	86.4	0.07
Family History (%)	14.3	5.8	2.4	5.6	0.35
Cancer (%)	21	17.7	21.6	35.7	0.36
Aspirin (%)	75	74.7	82.5	70.6	0.67
Clopidogrel (%)	0.0	6.2	4.9	8.8	0.72
Coumadin (%)	25.0	7.4	12.2	20.6	0.07
Nitrates (%)	20.0	22.2	12.5	11.8	0.44
Calcium antagonists (%)	15.0	34.6	26.8	17.7	0.16
ACE inhibitors (%)	30.0	42.0	56.1	58.8	0.10
Beta-blockers (%)	50.0	51.9	68.3	76.5	0.05
Statins (%)	25.0	47.8	56.1	67.7	**0.02**

Abbreviations: AAA, abdominal aortic aneurysms; ACE, angiotensin converting enzyme; COPD, chronic obstructive pulmonary disease; EVAR, AAA with elective endovascular repair; HDL, high-density lipoprotein; LDL, low-density lipoprotein; NA, data not available; rAAA, ruptured AAA with open repair; eAAA, AAA with elective open repair; sAAA, small AAA under surveillance; SD, standard deviation.

Note: Laboratory results are shown as mean ± SD and were measured preoperatively.

a Blood pressure ≥ 140/90 mm Hg.

b Increased LDL (male and female: ≥ 4.12 mmol/L; age > 35 years) and decreased HDL (male: ≤ 0.90 mmol/L; female ≤ 1.3 mmol/L; age > 35 years).

**Table 6:** Comparison of each AAA group with the control group

Variable	rAAA versus control group	eAAA versus control group	EVAR versus control group	sAAA versus control group
	*p* -Value ( *n* = 21)	*p* -Value ( *n* = 90)	*p* -Value ( *n* = 42)	*p* -Value ( *n* = 39)
Age (y) at time of study Mean ± SD	**0.012**	**< 0.001**	**<0.001**	**0.002**
Males (%)	0.561	**0.026**	0.337	**0.004**
Height (cm)	0.099	0.267	0.670	0.551
Weight (kg)	0.061	0.343	0.238	0.293
Hemoglobin (mmol/L)	**<0.001**	**<0.001**	**<0.001**	**<0.001**
White blood cell count (× 10 ^9^ /L)	**0.004**	0.379	0.194	0.968
Thrombocyte count (× 10 ^9^ /L)	0.182	**0.025**	0.184	0.050
Glucose	**0.003**	0.104	0.231	0.123
C-reactive protein (mg/L)	**<0.001**	**0.008**	0.368	0.276
Cholesterol (mmol/L)	0.515	0.888	0.140	0.534
LDL (mmol/L)	0.630	0.801	0.237	0.867
HDL (mmol/L)	0.521	0.906	0.929	0.135
Triglycerides (mmol/L)	0.264	0.093	0.333	0.181
Creatinine (mmol/L)	0.075	**<0.001**	**0.004**	**0.009**
Hypertension (%)	0.014	**<0.001**	**<0.001**	**<0.001**
Peripheral arterial disease (%)	**<0.001**	**<0.001**	0.006	0.043
Coronary artery disease (%)	0.010	**<0.001**	**<0.001**	**<0.001**
Myocardial infarction (%)	1.000	0.090	0.064	0.054
Diabetes mellitus (%)	0.770	0.853	0.631	0.503
Dyslipidemia (%)	1.000	**0.001**	**<0.001**	**<0.001**
COPD (%)	**0.008**	**<0.001**	**0.001**	**<0.001**
Ever smoke (%)	**0.043**	0.044	**0.006**	**<0.001**
Aspirin (%)	**<0.001**	**<0.001**	**<0.001**	**<0.001**
Clopidogrel (%)	0.603	1.000	0.723	0.723
Coumadin (%)	**0.034**	1.000	0.051	0.347
Nitrates (%)	0.070	0.003	0.454	0.301
Calcium antagonists (%)	1.000	**0.015**	1.000	0.254
ACE inhibitors (%)	0.612	0.645	0.069	0.090
Beta-blockers (%)	0.809	0.450	**0.003**	0.024
Statins (%)	0.447	0.066	**0.001**	0.024

Abbreviations: AAA, abdominal aortic aneurysms; ACE, angiotensin converting enzyme; COPD, chronic obstructive pulmonary disease; EVAR, AAA with elective endovascular repair; HDL, high-density lipoprotein; LDL, low-density lipoprotein; rAAA, ruptured AAA with open repair; eAAA, AAA with elective open repair; sAAA, small AAA under surveillance; SD, standard deviation.

A total of 48 (25%) of the 195 AAA patients reported having had an inguinal hernia and 26 (23%) of the 112 patients who had undergone an open repair (rAAA + eAAA) reported an incisional hernia ( Table 7 ). Many of the patients with inguinal hernia (32/48, 67%) had had herniorrhaphy, increasing the likelihood that the diagnoses reported by the patients in the questionnaire were correct. The prevalence of inguinal hernia in the control group was significantly lower than in the AAA group (9/100, 9%, *p* = 0.001). An incisional hernia was found in 2 (2%) of control patients. We compared the prevalences of hernias in the different treatment groups for AAA ( Tables 7 and 8 ). The prevalences of inguinal and incisional hernias in the rAAA group of 22 patients were 9% (two patients) and 18% (four patients), respectively. Herniorrhaphy was reported in one patient with an inguinal hernia and one patient with an incisional hernia. In the eAAA group, 22 of 90 (24%) patients had inguinal hernia, 12 (55%) of which had been repaired operatively. In addition, 22 of 90 (24%) of the eAAA group patients reported incisional hernia, 11 (50%) of which were surgically repaired. In the EVAR group, 15 (35%) patients had inguinal hernias, 12 (80%) of which had been surgically repaired. In the sAAA group, 9 of 40 (23%) patients had a history of inguinal hernias, 7 (78%) of whom reported having had a herniorrhaphy.

**Table 7:** Prevalences of different types of hernias of the abdominal wall among abdominal aortic aneurysms patients in the vascular center of the University Hospital Carl Gustav Carus, Technical University of Dresden, Germany

Group	AAA patients, *n* (%) total number *n* (%)	AAA patients, *n* (%) with incisional hernia after AAA surgery *n* (%)	AAA patients, *n* (%) with incisional hernia after other surgeries *n* (%)	AAA patients, *n* (%) with inguinal hernia *n* (%)
*rAAA* :				
All	22	4 (18)	1 (4.6)	2 (9.1)
Male	19 (86)	4 (21)	1 (5.3)	2 (10.5)
Female	3 (14)	0	0	0
*eAAA* :				
All	90	22 (24)	5 (6.8)	22 (24)
Male	82 (91)	21 (25)	4 (5.5)	21 (25)
Female	8 (8.9)	1	1 (17)	1 (13)
*EVAR* :				
All	43		3 (7.0)	15 (35)
Male	42 (98)		3 (7.1)	15 (36)
Female	1 (2.3)		0	0
*sAAA* :				
All	40		2 (5.0)	9 (23)
Male	35 (88)		2 (5.7)	9 (26)
Female	5 (13)		0	0
*All AAA cases* :				
All	195	26 (23)	11 (5.6)	48 (25)
Male	178 (91)	25 (13)	10 (5.6)	47 (26)
Female	17 (9)	1 (0.005)	1 (5.9)	1 (5.9)
*Control group* :				
All	100		2 (2)	9 (9)
Male	79 (79)		1 (1)	6 (6)
Female	21 (21)		1 (1)	3 (3)

Abbreviations: AAA, abdominal aortic aneurysms; EVAR, AAA with endovascular repair; rAAA, ruptured AAA; eAAA, AAA with elective open repair; sAAA, small AAA under surveillance.

**Table 8:** Comparison of different abdominal aortic aneurysms treatment groups for the presence of inguinal and incisional hernias

Hernia type	Comparison of AAA and control groups	*p* -Value a	OR (95% CI)
Inguinal	EVAR versus rAAA	**0.040**	5.1 (1.01–25.3)
	sAAA versus rAAA	0.840	2.73 (0.52–14.3)
	eAAA versus rAAA	0.560	3.02 (0.64–14.24)
	sAAA versus procedure (rAAA + eAAA + EVAR)	0.730	0.86 (0.37–2.0)
	EVAR versus open repair (rAAA + eAAA)	0.110	1.96 (0.87–4.37)
	sAAA versus open repair (rAAA + eAAA)	0.530	1.06 (0.34–2.56)
	All AAA versus control	**0.006**	4.00 (1.49–10.66)
Incisional	eAAA versus rAAA	0.390	1.69 (0.51–5.55)

Abbreviations: AAA, abdominal aortic aneurysms; CI, confidence interval; EVAR, AAA with endovascular repair; OR, odds ratio; rAAA, ruptured AAA; eAAA, AAA with elective open repair; sAAA, small AAA under surveillance.

a Statistical analysis was performed with multivariate logistic regression adjusted for sex and age.

Comparisons between males and females for family history of AAA ( *p* = 0.79; odds ratio [OR] = 0.84; 95% confidence interval [CI]: 0.10–7.11), incisional ( *p* = 0.24; OR = 3.0; 95% CI: 0.38–23.44), or inguinal hernias ( *p* = 0.16; OR = 2.61; 95% CI: 0.57–11.87) were tested with univariate analysis and were not statistically significant. Only one female patient had an incisional hernia, and only two females had inguinal hernias, which could explain the lack of significance, as well as the wide CIs. To determine if the female patients were influencing the statistical outcomes, we ran additional analyses on the male patients only. The results were similar to those of the entire group and are included here. When all four groups were compared for the prevalence of inguinal hernias in univariate analysis, there was no significant difference between the groups ( *p* = 0.15 for the entire sample, and *p* = 0.21 for males only).

Prevalence of inguinal hernias did not differ ( *p* = 0.15; OR = 3.2; 95% CI: 0.7–14.96 for the entire sample, and *p* = 0.23; OR = 2.7; 95% CI: 0.6–12.91 for males only) between the two open surgery groups, rAAA and eAAA, nor was there a difference when comparing all three procedures (rAAA, eAAA, or EVAR) to surveillance (sAAA; *p* = 0.73; OR = 0.86; 95% CI: 0.38–2.0 for the entire sample, and *p* = 0.98; OR = 0.99; 95% CI: 0.43–2.3 for males only).

No difference ( *p* = 0.21 for the entire sample, and *p* = 0.22 for males only) in the prevalence of inguinal hernias existed between those patient groups which had open surgery (rAAA and eAAA) compared with those with a minimally invasive procedure (EVAR) or surveillance (sAAA).

All groups were then compared using multivariate analysis, controlling for the probable confounding factors of age and sex ( Table 8 ). For the prevalence of inguinal hernia, the comparison of all AAA patients versus control patients was highly significant ( *p* = 0.006; OR = 4.00; 95% CI: 1.49–10.66), while EVAR versus rAAA was borderline significant ( *p* = 0.04; OR = 5.1; 95% CI: 1.01–25.3). The other groups were not statistically different from each other.

Only the surgical groups (rAAA compared with eAAA) were analyzed for incisional hernias, and no significant difference was found ( *p* = 0.39).

## Discussion

Our study demonstrated a higher prevalence of inguinal hernia in AAA patients compared with the control group and a significant association between hernia and AAA in a multivariate analysis. The prevalence of inguinal hernia did not, however, differ between the AAA subgroups, suggesting that hernias do not contribute to increased severity of the aneurysmal disease.

Patients diagnosed with known connective tissue disorders such as the Marfan syndrome (OMIM ID: 154700), the Loeys–Dietz syndrome (OMIM IDs: 609192, 613795, 608967, 610380, 614816, and 610168), and the Ehlers–Danlos vascular type (OMIM ID: 130050) are prone to aortic aneurysms and dissections, as well as to rupture of other hollow organs such as uterus and bowel ( http://omim.org ). They are also more susceptible to inguinal and incisional hernias. These patients have mutations in genes of the structural proteins of the connective tissues, that is, fibrillin I ( *FBN1* ) in the Marfan syndrome, in the *SMAD3* , *TGFB2* , and TGFB-receptor genes ( *TGFBR1* and *TGFBR2* ) in the syndromic forms of thoracic aortic aneurysms, and in Type III collagen gene ( *COL3A1* ) in the case of Ehlers–Danlos syndrome Type IV, also known as the vascular type of the Ehlers–Danlos syndrome.

It is possible that more common nonsyndromic diseases, such as AAAs also involve a connective tissue defect 3 which increases the patient's risk for abdominal wall hernias. Although AAA and abdominal wall hernias present common risk factors (e.g., smoking), there seems to be an independent association of these two diseases. 3 5 Clinical observations show that abdominal wall hernias are more common in AAA patients than in the general population. 9 Furthermore, pathobiological studies on abdominal wall hernias revealed similarities to the pathogenesis of AAA. 11 12 13 14 15

Several studies have investigated the expression of proteases responsible for degradation of the extracellular matrix (ECM). Matrix metalloproteinase 1 (MMP1) expression was increased in scar tissue of the skin from patients with incisional hernia and recurrent inguinal hernias, 14 but not in patients with primary inguinal hernia. 14 Similarly, elevated levels of MMP1 have been detected in patients with AAA. 15 Also, fibroblast cultures from the abdominal skin in direct inguinal hernia patients showed an increased matrix metalloproteinase 2 (MMP2) expression, 13 MMP2 mRNA levels were increased in incisional hernia samples, 14 and in serum of AAA patients. 15

The expression of metalloproteinase inhibitor 2 (TIMP2), one of the naturally occurring inhibitors of MMPs, was decreased in abdominal skin of hernia patients, 13 in fascia transversalis specimens of inguinal hernia patients, 11 and in the aneurysmal aortic wall. 15 These findings suggest an imbalance between the proteolytic activity of MMPs and their inhibitors in the abdominal skin prone to hernia formation and in the aortic wall with aneurysmal dilatation.

It is also plausible that hernias and AAAs develop due to defects in the structural proteins of skin, fascia, and aorta. The ratio of Type I to III collagen was shown to be reduced in skin fibroblasts, fascia transversalis, and the hernial sac in patients with inguinal and incisional hernia. 12 14 Collagens, which are major structural components of the ECM in the aorta and provide support and strength to the aorta, have also been shown to play a role in AAA development and rupture. 3

In conclusion, the results summarized above provide strong evidence for the hypothesis that similar pathobiological mechanisms are involved in hernias and AAA. Consistent with this hypothesis are findings of two recently published studies. One of them detected an increased prevalence of AAA among 235 patients with inguinal hernia who were >55 years old compared with a control group of 203 patients without inguinal hernias. 16 Another group of investigators found an increased prevalence of abdominal wall hernia in 110 patients with AAA (45%) in comparison with 60 patients with AOD (5%), disease that shares many of the same risk factors ( *p* -value < 0,001). 17 Three other studies with fewer patients, however, showed contradictory results. 7 18 19

It has also been suggested that surgical operations increase the patient's risk for hernia formation. One study detected higher prevalence of inguinal hernias after appendectomies and suggested changes in the muscle strength of the abdominal wall after transperitoneal operation. 20 In our study, the prevalence of hernias was not increased among patients who had been operated using midline incision compared with AAA patients who were under surveillance. Moreover, a Danish nationwide retrospective study found that AAA is an independent risk factor for incisional hernia repair in patients undergoing open elective aortic reconstructive surgery. Patients undergoing AAA repair have a 1.6-fold higher risk of a subsequent hernia repair than patients undergoing AOD repair. 21

Several previous studies showed a high prevalence of inguinal and incisional hernias among AAA patients ( Tables 1 and 2 ). A total of 12 studies, 11 of which presented a comparison between AAA and control groups, have been published on the prevalence of inguinal hernias. In 8 of 11 of these studies, the prevalence of inguinal hernias among AAA patients was significantly higher than in the control group, and ranged between 19 and 41% ( Table 1 ). In three of the studies ( Table 1 ), the prevalence of inguinal hernia was 14, 16 and 22% in the AAA group compared to 16, 9, and 17% in the control group, respectively. Only two previous studies investigated the prevalence of abdominal wall hernias in patients with large and small AAA (AAA < 55 vs. AAA > 55 mm). While Gindera et al 35 demonstrated a lack of association between the two diseases, which confirms the finding of our study, Pitoulias et al 17 found a significantly higher prevalence of hernia in large aneurysms (50 vs. 29.2%, *p* = 0.03). A recent study investigated the prevalence of AAA in patients with inguinal hernias and demonstrated a 27.7% prevalence of AAA among the study group. 22

The prevalence of incisional hernias has been reported in 19 studies, 12 of which presented a comparison between AAA and control groups ( Table 2 ). Only 7 of 12 studies found a significant difference in the prevalence of incisional hernias between AAA (range: 10–37%) and control (range: 3–26%) groups ( Table 2 ).

It is possible to minimize the risk for incisional hernia by surgical techniques, such as using a nonabsorbable or slowly absorbable material, 8 or prophylactic preperitoneal mesh placement after an open AAA repair. 5 8 Results with a retroperitoneal approach in AAA repair were inconsistent. 23 24 A meta-analysis published in 2007 described a 2.8-fold higher risk for incisional hernias (after midline transperitoneal approach) and 2.9-fold higher risk for inguinal hernias in patients with AAA. 6 The mean prevalence for inguinal and incisional hernias was 25 and 21%, respectively. 6 While the current study was in progress, Antoniou et al 5 and Takagi and Umemoto 25 repeated the meta-analysis, both with similar results.

### Limitations

One limitation of the current study is that we obtained information about hernias by a questionnaire sent to the AAA patients. It is well known that mailed questionnaires have potential biases, since it is not clear if the answers are based on a proper clinical evaluation, or patient's self-perception. Furthermore, physical examination of hernias is known to have a relatively poor diagnostic accuracy. 26 In the current study, information about herniorrhaphies performed in the past was also collected. Most of the patients who reported having inguinal hernias (67%), had had herniorrhaphy, suggesting that the information can be considered reliable and is not based on only patient's self-perception or physical examination. Despite the differences in the study designs, our results agree with the previous studies (25% for inguinal hernias and 23% for incisional hernias). Furthermore, another study, which also used a questionnaire, found that 31% of AAA patients had inguinal hernias, 4 a prevalence very similar to ours. Further, the prevalence of inguinal hernias in our control group was relatively low (9%) when compared with previous studies (5–27%).

Another limitation of the study is that the response rate of sAAA patients was lower (51%) than that of patients in the other groups (85–92%) who underwent operations. This could have influenced the results, since it is possible that patients with existing hernias were more motivated to answer the questionnaire than the ones without hernias. The results presented here should be considered preliminary, since the sample sizes of the study groups were small. Further, stratifying the severity of AAA disease by therapy type (rAAA vs. EVAR vs. eAAA vs. sAAA) might not be as accurate as exact measurements of the AAA size or stress mapping of the aneurysm wall tension. However, this approach gives a general idea of the disease severity without the need of further, sometimes complicated and time-consuming measurements, and can therefore be easily undertaken in clinical practice. Finally, approximately half of the patients with ruptured AAA died perioperatively and could not be studied here.

## Conclusions

In conclusion, our study confirms previous observations that patients with AAA have a relatively high prevalence of hernias and showed that the prevalence of hernias among AAA patients was not correlated with the size or rupture risk of AAA. Furthermore, prevalence of hernias was not increased among patients who had been operated compared with AAA patients who were under surveillance suggesting that surgical repair operation is not responsible for the increased hernia prevalence among AAA patients. These results suggest that the occurrence of hernias is not associated with a more aggressive AAA disease.
